# Ensembl variation resources

**DOI:** 10.1186/1471-2164-11-293

**Published:** 2010-05-11

**Authors:** Yuan Chen, Fiona Cunningham, Daniel Rios, William M McLaren, James Smith, Bethan Pritchard, Giulietta M Spudich, Simon Brent, Eugene Kulesha, Pablo Marin-Garcia, Damian Smedley, Ewan Birney, Paul Flicek

**Affiliations:** 1 European Bioinformatics Institute, Wellcome Trust Genome Campus, Hinxton, Cambridge, CB10 1SD, UK; 2 Wellcome Trust Sanger Institute, Wellcome Trust Genome Campus, Hinxton, Cambridge, CB10 1SA, UK

## Abstract

**Background:**

The maturing field of genomics is rapidly increasing the number of sequenced genomes and producing more information from those previously sequenced. Much of this additional information is variation data derived from sampling multiple individuals of a given species with the goal of discovering new variants and characterising the population frequencies of the variants that are already known. These data have immense value for many studies, including those designed to understand evolution and connect genotype to phenotype. Maximising the utility of the data requires that it be stored in an accessible manner that facilitates the integration of variation data with other genome resources such as gene annotation and comparative genomics.

**Description:**

The Ensembl project provides comprehensive and integrated variation resources for a wide variety of chordate genomes. This paper provides a detailed description of the sources of data and the methods for creating the Ensembl variation databases. It also explores the utility of the information by explaining the range of query options available, from using interactive web displays, to online data mining tools and connecting directly to the data servers programmatically. It gives a good overview of the variation resources and future plans for expanding the variation data within Ensembl.

**Conclusions:**

Variation data is an important key to understanding the functional and phenotypic differences between individuals. The development of new sequencing and genotyping technologies is greatly increasing the amount of variation data known for almost all genomes. The Ensembl variation resources are integrated into the Ensembl genome browser and provide a comprehensive way to access this data in the context of a widely used genome bioinformatics system. All Ensembl data is freely available at http://www.ensembl.org and from the public MySQL database server at ensembldb.ensembl.org.

## Background

The amount of publicly available biological sequence data has been increasing exponentially over the last decade. In addition to the many reference genome sequences now available, variation data is being produced in significant quantities. These data fundamentally seek to extend our knowledge of the genome sequence from the concept of a single "reference" genome sequence, representing a single individual, to a more comprehensive understanding of the genomic diversity of entire species.

Today most variation data is produced in the context of large-scale genotyping assays or resequencing projects which focus either on the whole genome or selected functional regions of the genome such as protein coding regions, regulatory regions or sites of known disease mutations. One of the larger resources includes a comprehensive haplotype map of the human genome created by the International HapMap Project, based on DNA from 270 individuals from four populations [1]. The HapMap Project used array-based genotyping to assess markers with minor allele frequency (MAF) of greater than approximately 5%. Following on from this project, many of these same HapMap individuals are included with others in the 1000 Genomes Project [2], which seeks to assay variant sites including those with much lower MAFs. Previous efforts to map variation in other species include strain specific mouse resequencing [3,4], a haplotype mapping in rat [5], as well as data mining of public domain resources such as NCBI's dbSNP [6]. Whole genome shotgun sequencing can be used for reliable variant discovery in a single sequenced individual by comparing the sequencing reads to the final consensus assembly as done with platypus [7]. When a reference assembly exists, it is more efficient to compare the sequenced individual to that assembly as this technique facilitates the discovery of both heterozygous variants within the individual and variants between the individual and the reference. Reference based variation discovery has been used for several species including human [8,9], mouse [4], rat [5], and chicken [10].

These huge datasets including variation data are often available from the original sources in a variety of formats requiring the development of various methods to integrate, archive and display these data in a consistent fashion. Ensembl [11], University of California at Santa Cruz (UCSC) [12,13] and the National Center for Biotechnology Information (NCBI) [14] have expertise in the storage and manipulation of biological data and have developed genome browsers and other methods to archive and display these data alongside their other large scale data resources. The variation data stored in Ensembl are discussed here.

### Ensembl

The Ensembl project is a comprehensive bioinformatics resource for chordate genomes. Thousands of researchers from around the world access Ensembl data every day through the various portals provided by the project including the web interface at http://www.ensembl.org[15], the Ensembl API [16], and Ensembl BioMart [17]. In addition to the chordate genomes, selected model organisms (*D. melanogaster*, *C. elegans*, *S. cerevisiae*) are included to facilitate comparative analysis. More comparative analysis is available using species supported by Ensembl Genomes project, a sister project extending Ensembl analysis across a larger taxonomic space [18].

Ensembl is updated approximately every two months with newly sequenced genomes and newly available or processed data for existing genomes. The project specialises in integrating large-scale data from many different sources in a variety of formats with a high-quality annotation of the genome and gene set. In addition to comparative and functional genomics data resources, Ensembl provides variation data for a number of supported species.

Each year, Ensembl publishes a general update of all the project's resources [11,19,20]. In contrast to these high-level overviews, this is a more in-depth report specifically on the growing number of variation resources available within Ensembl. It describes, in detail, how data is extracted and combined from primary sources such as the Ensembl Trace Archive [21] and NCBI's dbSNP [6], how it is generated using data from resequencing information, how it is visualised and how to obtain the data via the website.

## Construction and Content

Ensembl produces variation databases for a subset of the genomes available in Ensembl (first two columns of Table 1). This allows integration and easy access to variation data from multiple sources as well as the effects of sequence variation on the genes. The databases incorporate four types of data: a complete polymorphism catalogue, genotype data from specific projects, phenotype data and selected resequencing data. The primary source of polymorphism and genotype data for SNPs and in-dels is from dbSNP, the major public archive of variation data, which is integrated with data from other sources as described below. Structural variants are imported from DGVa [22] and for some species, additional variants are generated from uniform processing and variation discovery using sequencing reads from the Ensembl Trace Archive (see column 4 of Table 1). Phenotype data comes from both a manually curated resource and a public archive, as explained below. Finally a series of quality-control stages are implemented as described below. In this way, Ensembl can begin to provide a comprehensive picture of variations, their effects, and their context.

**Table 1:** Variation statistics for release 56, September 2009 http://sep2009.archive.ensembl.org/index.html.

Name	Number of variants **	Genome size* (Assembly name)	Data source of variants
Homo sapiens	17,999,182	3,101,804,741 (GRCh37)	dbSNP; Resequencing reads

Pan troglodytes	1,520,077	3,350,417,645 (CHIMP2.1)	dbSNP

Pongo pygmaeus abelii	1,384,342	3,446,771,396 (PPYG2)	Assembly Reads

Mus musculus	14,888,174	2,716,965,481 (NCBIM37)	dbSNP; Resequencing reads

Rattus norvegicus	2,854,253	2,718,897,321 (RGSC3.4)	dbSNP; Resequencing reads

Canis familiaris	3,057,889	2,531,673,953 (BROADD2)	dbSNP

Bos taurus	2,057,872	2,918,205,644 (Btau_4.0)	dbSNP

Ornithorhynchus anatinus	1,207,507	2,073,148,626 (OANA5)	Assembly reads

Gallus gallus	2,960,841	1,100,480,441 (WASHUC2)	dbSNP

Danio rerio	617,481	1,481,257,891 (Zv8)	dbSNP

Tetraodon nigroviridis	903,588	358,618,246 (TETRAODON8)	Assembly reads

Taeniopygia guttata	4,745,545	1,233,169,488 (taeGut3.2.4)	Assembly reads

*Genome size refers to the length of the reference assembly.

**Distinct mapped variants

The Ensembl variation resources are updated when a new genome assembly is released, a new set of gene annotations is available or revised, an external data source such as dbSNP at the NCBI is updated or a major new data collection becomes available.

Variation data is stored in a structured MySQL database. A companion paper describes the underlying database technology that is used to efficiently store and manage this data and the associated Application Program Interface (API) for programmatic access to the data [23].

### Data Sources

#### Importing variation data from dbSNP

The bulk of the SNP and in-del variation data in Ensembl is an imported dataset from dbSNP. This information is selected from dbSNP's largest tables. Table 2 describes the relationship between the dbSNP database tables and the corresponding Ensembl variation database tables.

**Table 2:** List of Ensembl tables generated and dbSNP tables used

*Ensembl tables generated *	*dbSNP tables used *
source	

variation	SNP, SNPAncestralAllele

variation synonym*	SubSNP, SNPSubSNPLink, ObsVariation

	Batch, UniVariAllele

population/sample/population structure/sample synonym	PopClassCode, PopClass, PopLine

individual/sample/individual population/sample synonym	SubmittedIndividual, Individual PedigreeIndividual

allele	Allele, AlleleFreqBySsPop, SubSNP

flanking sequence	SubSNPSeq5, SubSNPSeq3, SNP

individual genotype	SubInd, ObsGenotype, SubmittedIndividual

population genotype	GtyFreqBySsPop, UniGty, Allele

variation feature	SNPContigLoc, ContigInfo

Normally, the reference genome assembly used by Ensembl matches the one used in the current build of dbSNP. When this is the case, the variant mapping positions can be imported directly from dbSNP and are therefore in sync with the rest of the data in Ensembl. As the genome browser at UCSC also uses the dbSNP variant mappings, using the same assembly is good for consistency across the major genome browsers.

In some cases however, the current genome assembly supported by Ensembl is ahead of the one used in the most recent build of dbSNP. When this occurs, the variants and their flanking sequences need to be remapped to the new assembly using a method in the core Ensembl API. For those variants that fail to map, new mappings are obtained using ssaha2, an improved version of SSAHA http://www.sanger.ac.uk/resources/software/ssaha2/ [24]. The flanking sequence is aligned with the parameters *kmer *= 12 *seed *= 4 *cut *= 1000 *depth *= 5 *best *= 1. For species with shorter flanking sequences, such as the zebrafish, *seed *= 2 is used.

The effect of this procedure is to ensure that dbSNP variation data in Ensembl is mapped to the most recent genome assembly supported by Ensembl, even if the assembly is not yet supported by dbSNP. As noted above, when the new dbSNP build is released, Ensembl is updated with the dbSNP defined variant positions. For a very small number of SNPs, this dbSNP update will result in changes to the SNP locations that had been defined by the Ensembl mappings on the new assembly. These changes are due to the method of incorporation of newly submitted data by reclustering of the variants as part of each dbSNP build.

Genotype data is imported from dbSNP and integrated with the polymorphism data described above. For example, in release 56 Ensembl has genotype data for 8335 individuals from a number of reference data sets including the HapMap [25], Perlegen [26] and the 1000 Genomes Project [2].

#### Array Data: Illumina, Affymetrix

Primary identifiers are assigned to variations. Usually these are from dbSNP (rs and ss identifiers). However, there may be other sources for variations. Names in these other datasets are only used as primary IDs if there is no matching dbSNP record. In the case of a dbSNP record, the IDs from other datasets are included as synonyms.

As of release 56 (September 2009), Ensembl imports variants positions from the following arrays: Affymetrix GeneChip Human Mapping 100 K Array Set, Affymetrix GeneChip Human Mapping 500 K Array Set, Affymetrix Genome-Wide Human SNP array 6.0, Illumina whole genome SNP genotyping chips designed for association studies (Human 660W-Quad, Human 1M-Duo V3) and for cytogenetic analysis (Cyto SNP-12 v1).

#### Legacy variation identifiers

Previously, Ensembl provided the identification page numbers from The SNP Consortium [9] and HGVBase [27] as synonyms to the primary variation identifiers. These identification numbers were prefaced with codes TSC and SNP respectively. As neither of these databases is active the identification numbers have been removed from the current database but are still accessible via the Ensembl Archive websites http://www.ensembl.org/info/website/archives/index.html and available by FTP ftp://ftp.ebi.ac.uk/pub/software/ensembl/snp/.

#### Phenotype data

Ensembl currently imports phenotype data from two sources: The National Human Genome Research Institute's (NHGRI) Office of Population Genomics and from the European Genome-phenome Archive (EGA) database [28].

NHGRI has developed a curated database of published significant genomic regions identified from genome-wide association studies http://www.genome.gov/GWAstudies[29]. More regions are being added as additional GWAS studies are published. These data are updated for every release of Ensembl.

The EGA database at the European Bioinformatics Institute is designed to provide a permanent archive for all types of personally identifiable genetic data including genotypes, genome sequence, and associated phenotype data.

In Ensembl release 56 (September 2009), data from these two resources total 134 phenotypes from 1120 possible phenotype annotations associated with 142 different variants. There is a direct link from the web page to the original submitter's data entry under the "Source" column in order to acknowledge the contributor directly. In addition a link is provided to the publication record in PubMed.

### Data generation

#### Algorithm for Variant discovery from Sequencing reads

Ensembl provides the location of specific variants in individuals, laboratory strains and breeds. These variants are calculated as described here using publicly available Sanger-style resequencing data such as from the Ensembl Trace Archive or from whole genome projects that sequence a single individual (with both haplotypes) or small number of individuals. By aligning the sequencing reads to the reference assemblies for the same species, Ensembl is able to identify any heterozygous sites in the sequenced individual by mismatches in the alignment [4]. These alignments are determined using the ssahaSNP pipeline http://www.sanger.ac.uk/resources/software/ssahasnp/[24] and require both the sequence data and sequencing quality PHRED scores in order to ensure high quality. Using the neighbourhood quality score (NQS) method [30], SNPs were called from alignments only when the variant base in the sequencing read had PHRED quality value greater than or equal to 23, the five bases on each side of the variant aligned exactly to the assembly with PHRED quality scores greater than 15 and when there was no more than one mismatch in 10 flanking base pairs.

Furthermore, potential variants are filtered and discarded if they have more than 30 mismatches from a single sequence read or are in a region with more than 100 aligned reads. (These values are reduced if the sequencing coverage is less than approximately 0.1×). Read pair information is used to filter out wrongly mapped reads, and variants with more than two alleles for one strain/individual are discarded. For individuals or strains with a read coverage of greater than 3, a SNP will be reported if at least two reads have the minor allele.

Ensembl also examines the resequencing reads that align to known SNP loci in order to verify the sequence of the resequenced individual at these locations. If there is sufficient evidence (defined with the same quality metrics as above) to determine that the given individual does not have an alternative allele at the tested location, this information is stored in the database as a "SARA" (Same As Reference Assembly) position, which can be thought of as a computational genotyping assay.

A representation of the resequencing coverage for several mouse and rat strains as well as the Watson and Venter genome sequences is stored in the variation database as a range of start and end coordinates with an indication of the level of read coverage.

The variation database generated by Ensembl's SNP calling pipeline is then merged with the variation database imported from dbSNP. Part of the merging procedure involves modifying all of the dbSNP imported variants to represent them on the positive strand. A variant by Ensembl from resequencing data that has the same locus as one already in dbSNP is assigned the rsID from dbSNP for that locus as the primary ID; those not yet in dbSNP can be identified by the Ensembl style identifiers and are submitted by Ensembl to dbSNP.

### Data quality checks

Once the database has been populated with variation data, a series of post processing steps is performed to ensure data consistency and quality. Variants observed multiple times (e.g. in data imported from dbSNP and created by analysis of the Ensembl Trace Archive) are collapsed into a single record with supporting identifiers stored as synonyms.

Variants are considered unreliable and flagged as "failed" if they exhibit any of the following characteristics: variants which map more than three times; those which do not map to the genome; those with no observed allele matching the reference allele; those with more than three observed alleles. Failed variants remain in the database but have no associated mapping position. There is also a checklist to ensure those medically important or other known to be correct variants that exhibit one of the above characteristics are not moved inadvertently to the failed variation table. Approximately 689660 variants are classified as failed across all supported species in release 56 (September 2009) (see Table 3).

**Table 3:** Breakdown of failed variants by type for each species

Name	Type 1	Type 2	Type 3	Type 4	Type 5	Total number
**Homo sapiens**	132,037	494,262	37,456	3,359	13,768	680,882

**Pan troglodytes**	0	0	7	0	2,885	2,892

**Pongo pygmaeus abelii**	0	0	0	0	0	0

**Mus musculus**	155,456	299,923	0	0	25,691	481,070

**Rattus norvegicus**	1,475	482	1	0	216	2174

**Canis familiaris**	27,518	15,660	55	0	11,986	55,219

**Bos taurus**	9,679	103,072	0	0	490	113,241

**Ornithorhynchus anatinus**	0	4849	0	0	0	4,849

**Gallus gallus**	11,070	88,558	0	0	229,878	329,506

**Danio rerio**	0	0	0	0	44,841	44,841

**Tetraodon nigroviridis**	0	0	0	0	0	0

**Taeniopygia guttata**	0	0	0	0	0	0

Type 1: Variant maps to more than 3 different locations

Type 2: None of the variant alleles match the reference allele

Type 3: Variant has more than 3 different alleles

Type 4: Loci with no observed variant alleles in dbSNP

Type 5: Variant does not map to the genome

### Variation consequence annotation

Once a list of reliable variants has been identified, they are integrated with the Ensembl gene annotations in order to estimate the consequence of each variant on all transcripts. A list of possible consequences assigned in shown in Table 4. This section of the pipeline is recalculated whenever the gene sets for a species is updated.

**Table 4:** List of consequence types in the transcript variation table

Consequence type	Effect	Sequence location
Stop gained	causes a gain of a stop codon	coding sequence of the peptide

Stop lost	causes a loss of a stop codon	coding sequence of the peptide

complex InDel	change in nucleic acid	Indel spanning exon/intron or CDS/UTR border

Frameshift coding	causes a frameshift of the reading frame	affects the coding sequence of the peptide

Stop gained, frameshift coding	multi-allelic variant that introduces a stop codon or causes a frameshift	affects the coding sequence

Non-synonymous coding	causes an amino acid change	coding sequence of the peptide

Stop gained, splice site	causes a gain of a stop codon	1-3 bps into an exon

Stop lost, splice site	causes a loss of a stop codon	1-3 bps into an exon

Frameshift coding, splice site	causes a frameshift	1-3 bps into an exon

stop gained, Frameshift coding, splice site	Multi-allelic variant that introduces a stop codon or causes a frameshift	1-3 bps into an exon

Non-Synonymous coding, splice site	causes an amino acid change	1-3 bps into an exon

Synonymous coding	change in nucleic acid but no change in amino acid	coding sequence of the peptide

Splice site, synonymous coding	change in nucleic acid but no change in amino acid	1-3 bps into an exon

Regulatory region	change in nucleic acid	regulatory region annotated by Ensembl

Within mature miRNA	change in nucleic acid	within mature miRNA

5' UTR	change in nucleic acid	In 5' UTR

Splice site, 5' UTR	change in nucleic acid	1-3 bps into a 5' UTR exon

3' UTR	change in nucleic acid	In 3' UTR

Splice site, 3' UTR	change in nucleic acid	1-3 bps into a 3' UTR exon

Intronic	change in nucleic acid	In intron

Essential splice site, intronic	change in nucleic acid	in the first 2 or the last 2 base pairs of an intron

Splice site, intronic	change in nucleic acid	3-8 bps into an intron

Upstream	change in nucleic acid	Within 5 kb upstream of the 5'-end of a transcript

Downstream	change in nucleic acid	Within 5 kb downstream of the 3'-end of a transcript

Within non-coding gene	change in nucleic acid	Within non-coding gene

Intergenic	change in nucleic acid	More than 5 kb away from a transcript

Variants are associated with transcripts if they are located within transcript or intron sequences, within 5 KB of a transcript start or end, or if they are located within a regulatory region associated with a gene or transcript. Ensembl ranks these "consequence types" in estimated order of importance, including any effect of the variant on the sequence of the final protein product. For example ESSENTIAL SPLICE SITE is considered one of the most important consequence types as it would have great affect on the protein product.

### Calculation of Linkage Disequilibrium (LD)

The method used to calculate LD (r^2 ^and D') is described below. For each population with a sample size of at least 40, variants are ordered by their positions and pairwise LD (r^2 ^values) are calculated within a 100 kb window size. If r^2 ^values are less than 0.05 they are discarded and the rest are stored in a table. This table is only used for the calculation of tagged SNPs and is not present in the final database. The r^2 ^and D' values used in Ensembl web interface are calculated separately on demand [23].

### Calculation of tagged SNPs

A list of tagged variants for haplotype analysis is produced for each of the HapMap and Perlegen populations and stored in the database. This is designed to choose common variants in each region of the genome (i.e. tag SNPs) from which the genotypes of surrounding SNPs in high LD can be derived. In order to store a minimal list, variants are selected per population and any other variants in high LD with this one are filtered out. To do this, variants that have genotypes are sorted by start position. For each variant, the MAF is calculated by population and then these are ordered by frequency with the highest MAF first. For each ordered variant, the pairwise r^2 ^value for all variants within a 100 KB is extracted from the LD table described above. If the r^2 ^value between the variations is greater than 0.99 (i.e. the two variants are in high LD), the associated variant with the lower MAF is removed from the list. This process removes variation data in high LD with other variants that have lower MAF. The remaining variations are called tagged SNPs and are stored in the database.

## Utility and access of variation data

As with all Ensembl databases, the data is accessible in multiple ways: either programmatically via the Ensembl-style Application Program Interface (API) [23], or online using the Ensembl genome browser visualisation tools and the Ensembl BioMart tool. Ensembl's variation specific web displays, along with a variation focused BioMart query, are described in detail below. To facilitate bioinformatics analysis, the Ensembl databases are publicly available at the MySQL database server on ensembldb.ensembl.org and the BioMart databases at martdb.ensembl.org.

### Data Visualisation

Ensembl provides a number of specialised and highly interactive web displays focusing on four levels: a genomic location, a gene, a transcript and a variation. These four types of view support distinct pathways into the data (Table 5), and are reflected by the four main tabs in the Ensembl interface. This allows researchers to explore complementary information from various perspectives. It is possible to customise most views in Ensembl using the "configure this page" link at the left of the web pages. The first tab considered, the variation tab, is available for all species with variation data within Ensembl (see the first column of Table 1).

**Table 5:** Variation Displays in the Ensembl website

Browser Object	Sub Panel
Location	resequencing data
	linkage data

Gene	variation table
	variation image

Transcript	population comparison
	comparison image

Variation	summary
	gene/transcript
	population genetics
	individual genotypes
	context
	phenotype data
	phylogenetic context

#### The variation tab

Consider the variation rs2476601, which has been well-studied (see for example http://www.snpedia.com/index.php?title=Rs2476601) and has been implicated in rheumatoid arthritis [31], among other diseases. Entering rs2476601 in the search box at http://www.ensembl.org returns a link to the variation tab, described below. A search for a disease or phenotype will also link to the variation views.

**Figure 1:**
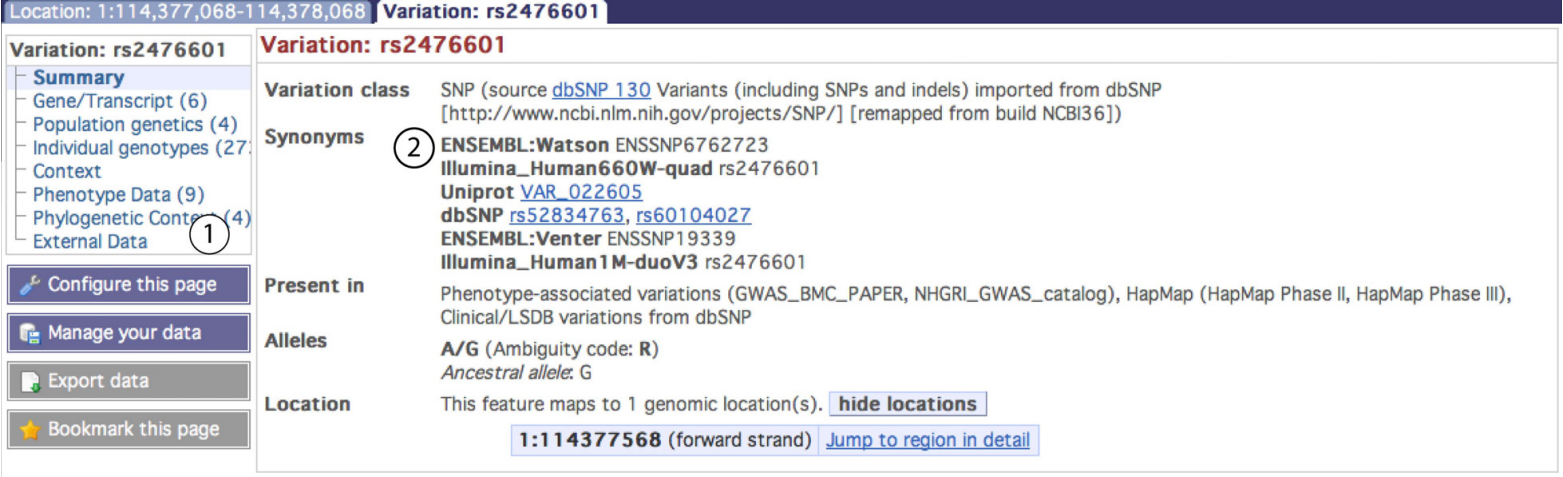
**Variation summary for rs2476601**. Label 1 indicates the links to information for this specific variant. Label 2 indicates the summary, which includes the source (dbSNP), synonyms or other IDs for this variant in other databases or array platforms, alleles found and genomic location. Flanking sequence is also found in the *summary *information but not shown in the figure. http://Mar2010.archive.ensembl.org/Homo_sapiens/Variation/Summary?source=dbSNP;v=rs2476601.

The variation tab contains data reports for one specific variation. A menu of links to these reports is available at the left hand side of the variation tab Figure 1**, label 1**. The variation identifier at the top of the variation summary is the dbSNP rs identifier, if one exists, otherwise the ss identifier or local identifier is chosen. Where appropriate, there is a link to the corresponding variation page in the dbSNP browser. The synonyms field (Figure 1, **label 2**) shows additional names for the variant, for example dbSNP ss IDs and IDs from other sources including Affymetrix SNP arrays and Illumina platforms, as described above. Flanking sequence, as reported by the dbSNP database, is shown on the page as are links to LD (linkage disequilibrium) plots, if available.

##### LD plots

**Figure 2:**
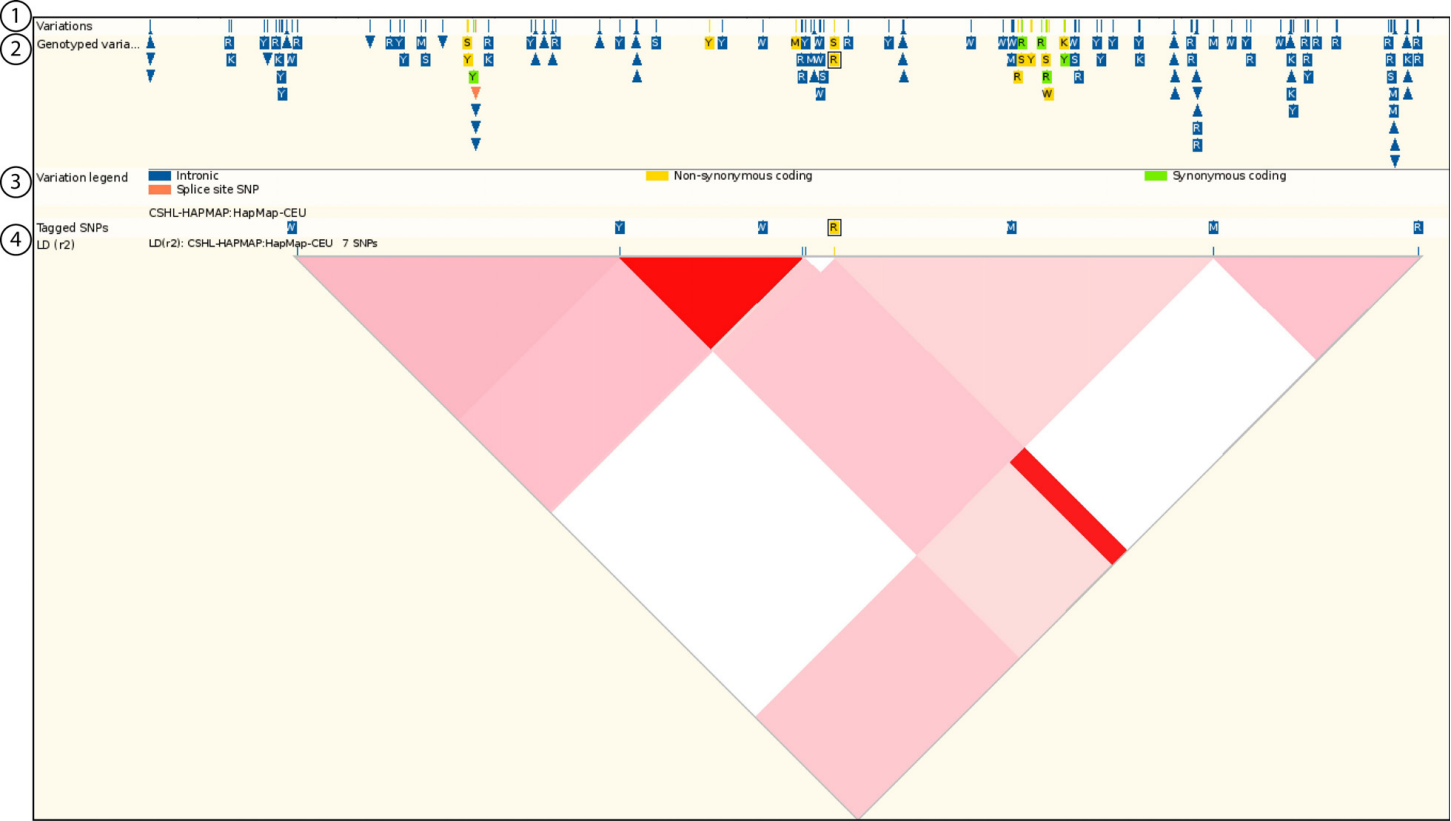
**LD (linkage disequilibrium) plot for the region around rs2476601**. LD values in this figure were calculated based on allele frequencies in the CEPH human population. The LD data between variants is represented using a triangular grid shaded on a gradient from white to red depending on the strength of the LD (where red is high LD, white is low). Hovering the mouse cursor over one of the coloured regions in the plot reveals a pop-up box displaying the two variation IDs for that coloured region, and the LD value between them. http://Mar2010.archive.ensembl.org/Homo_sapiens/Location/LD?focus=variation;pop1=CSHL-HAPMAP:HapMap-CEU;r=1:114367568-114387567;v=rs2476601;vf=1916990.

Following the link to an LD plot displays the LD data calculated within a region (Figure 2). The variation sources represented by the LD plot are customisable. Below the transcript diagrams there is a track showing all variations (Figure 2, **label 1**), a track showing only genotyped variations **(label 2) **and a track for tagged variants (**label 4**). Using the conventions described above and listed in Table 4, these tracks are colour-coded according to their location/effect on the transcript (**label 3**). Below this are LD *r*^2 ^and LD *D' *plots. The data for these plots, as described above, is calculated on-the-fly using a standard EM algorithm for calculating LD. The *export data *link at the left of the page will generated downloadable files of pairwise *r*^2 ^and *D' *values along with rs IDs, in HTML, text, or Microsoft Excel format. The data can also be exported in a format for uploading into Haploview software [32].

##### Variation links

**Figure 3:**
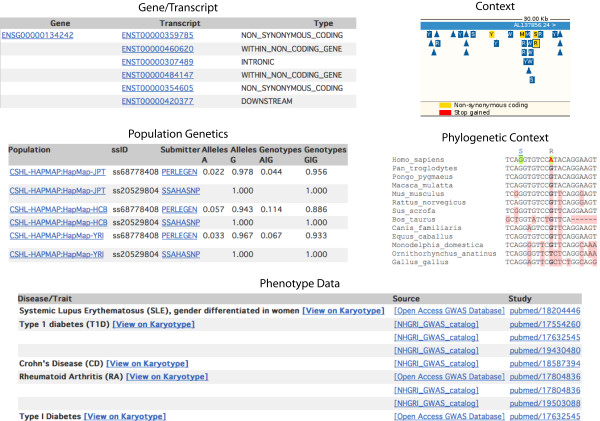
**Variation views**. Views available in the variation tab are titled with the name of the link leading to each set of information. The data in the figure correspond to rs2476601. The *gene/transcript *panel shows an Ensembl gene with this variant where it has different effects depending on the splice variant. Allele frequencies have been measured in four HapMap populations, as shown in the *population genetics *panel. The NHGRI associates Crohn's disease, Rheumatoid Arthritis and Type I Diabetes to this SNP, displayed in *phenotype data*. The *phylogenetic context *panel shows a common allele at this position in the reference sequences for chimpanzee (*Pan troglodytes*), orangutan (*Pongo pygmaeus*), macaque (*Macaca mulatta*), mouse (*Mus musculus*), rat (*Rattus norvegicus*), horse (*Equus caballus*), dog (*Canis familiaris*), cow (*Bos taurus*), opossum (*Monodelphis domestica*), and chicken (*Gallus gallus*). Platypus (*Ornithorhynchus anatinus*) shows a different nucleotide (T).

Links at the left of the page (Figure 1, **label 1**) are to views that provide information about the variation summary, the genes or transcripts in Ensembl associated with this variation, population allele and genotype frequencies imported from dbSNP, individual genotypes, a graphical view of the variation and neighbouring variations (the *context *link), phenotype data from the manually curated NHGRI GWAS catalogue and European Genome- phenome Archive http://www.ebi.ac.uk/ega/, and the phylogenetic context of the variant showing the allele at this position across species (including calculated Ensembl ancestral alleles) (Figure 3).

##### Gene/Transcript link

A table displays any transcript in Ensembl associated with the variant. The predicted effect of the variant is listed (e.g. intronic, synonymous coding, etc.) along with the position of the allele in the spliced transcript and protein. These values can be exported using BioMart.

##### Population genetics

Data from projects such as The HapMap Project or Perlegen are included in this view. Allele frequencies are displayed for specific populations, along with a summary of genotype information.

##### Individual genotypes

This next view shows a breakdown of genotypes for each individual stored in the database.

##### Context

The variant of interest is shown by default in a 30 Kb region with neighbouring SNPs as boxes and indels as triangles. Variation colour-coding is consistent throughout the Ensembl website and depend on the variant's localisation within a transcript (table 3).

##### Phenotype data

Listed in tabular format are any associated diseases, appropriate study and PubMed references, associated genes, the highest-risk allele and associated variant.

##### Phylogenetic context

This view displays a section from a whole-genome, multi-species alignment in this region. These alignments, including the calculated ancestral sequences are produced by Ensembl's three step Enredo-Pecan-Ortheus (EPO) pipeline [33,34]. Briefly, the process consists first of Enredo, which produces the initial collinear segments that appropriately take into consideration rearrangements, deletions and duplications from the genomes to be aligned. In the second step, the consistency-based method Pecan aligns the sets of sequences identified by Enredo taking into account the phylogenetic tree of species to be aligned. Finally, Ortheus uses a probabilistic transducer model to create genome-wide ancestral sequence reconstructions.

#### The gene tab

##### Genetic variation: table and image

**Figure 4:**
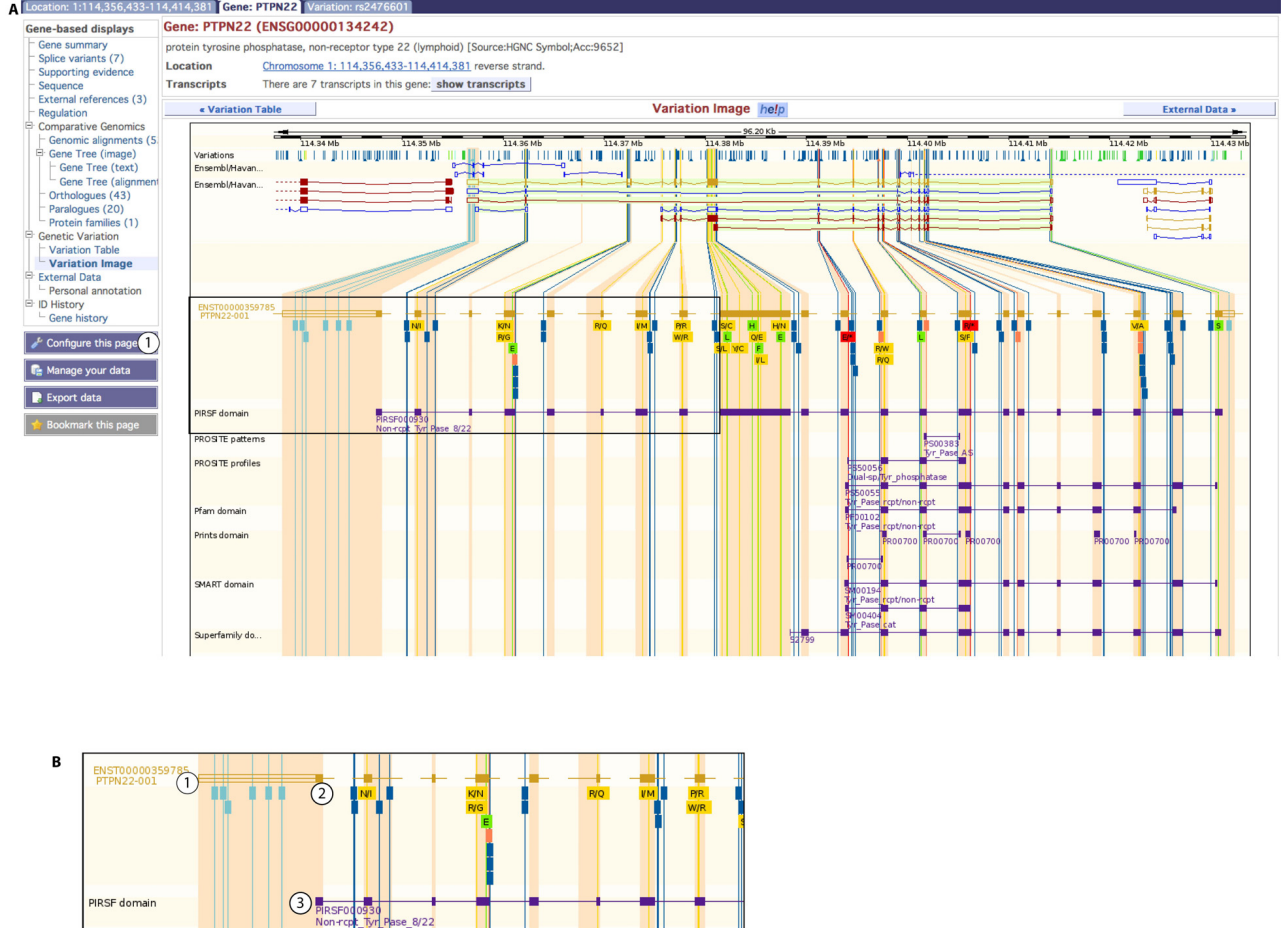
**Variation image**. **A**. The variation image in the gene tab shows all transcripts for an Ensembl gene in red, and variations mapping to each transcript as coloured boxes. Intronic variants are, by default, only drawn if within 100 bp of an exon. Change this by clicking on *configure this page *and selecting full introns. Protein domains and motifs encoded by the transcript sequence from databases such as PIR-Superfamily, PROSITE and Pfam are drawn in purple along each transcript. **B**. An expanded view of the box drawn in **A **is shown. Label 1 indicates the exon structure of Ensembl transcript ENST00000359785. Filled boxes are coding sequence within the exons, and unfilled boxes indicate untranslated regions (UTRs). Variations are indicated by label 2, and are colour-coded as to their effect on the transcript (see legend on the page). For this transcript, many intronic (dark blue) variations are shown, along with four non-synonymous (yellow) SNPs that display the potential amino acids at each position. Label 3 indicates a PIR-Superfamily domain (purple) that maps to the protein sequence. http://Mar2010.archive.ensembl.org/Homo_sapiens/Gene/Variation_Gene?db=core;g=ENSG00000134242;r=1:114356437-114414375;t=ENST00000359785.

Clicking on an Ensembl gene identifier or searching for a gene in the Ensembl search box (e.g. ENSG00000134242) moves the display to the gene tab. The gene tab provides links to the genetic *variation table *and the genetic *variation image*, which display the collection of known variation for a gene in a given species (Figure 4A, **label 1**).

In the *variation table*, each transcript (i.e. splice isoform) for the specific gene is shown, along with any associated variation. Effect on the transcript (if any), position in genomic coordinates, alleles, amino acids encoded and amino acid coordinates (if any) are listed. The source (or sources) of the variation and validation status are also shown.

The *variation image *(Figure 4) provides a graphical representation of this information. The addition of protein domains mapped to the amino acid sequences makes it possible to estimate any effect of a variation on protein structure and/or function.

##### Gene sequence

The genomic sequence can be configured to highlight variant positions. Exons are highlighted in this view, allowing immediate visualisation of where a variation falls, in the context of a gene.

#### The transcript tab

##### Transcript variation: table and image

**Figure 5:**
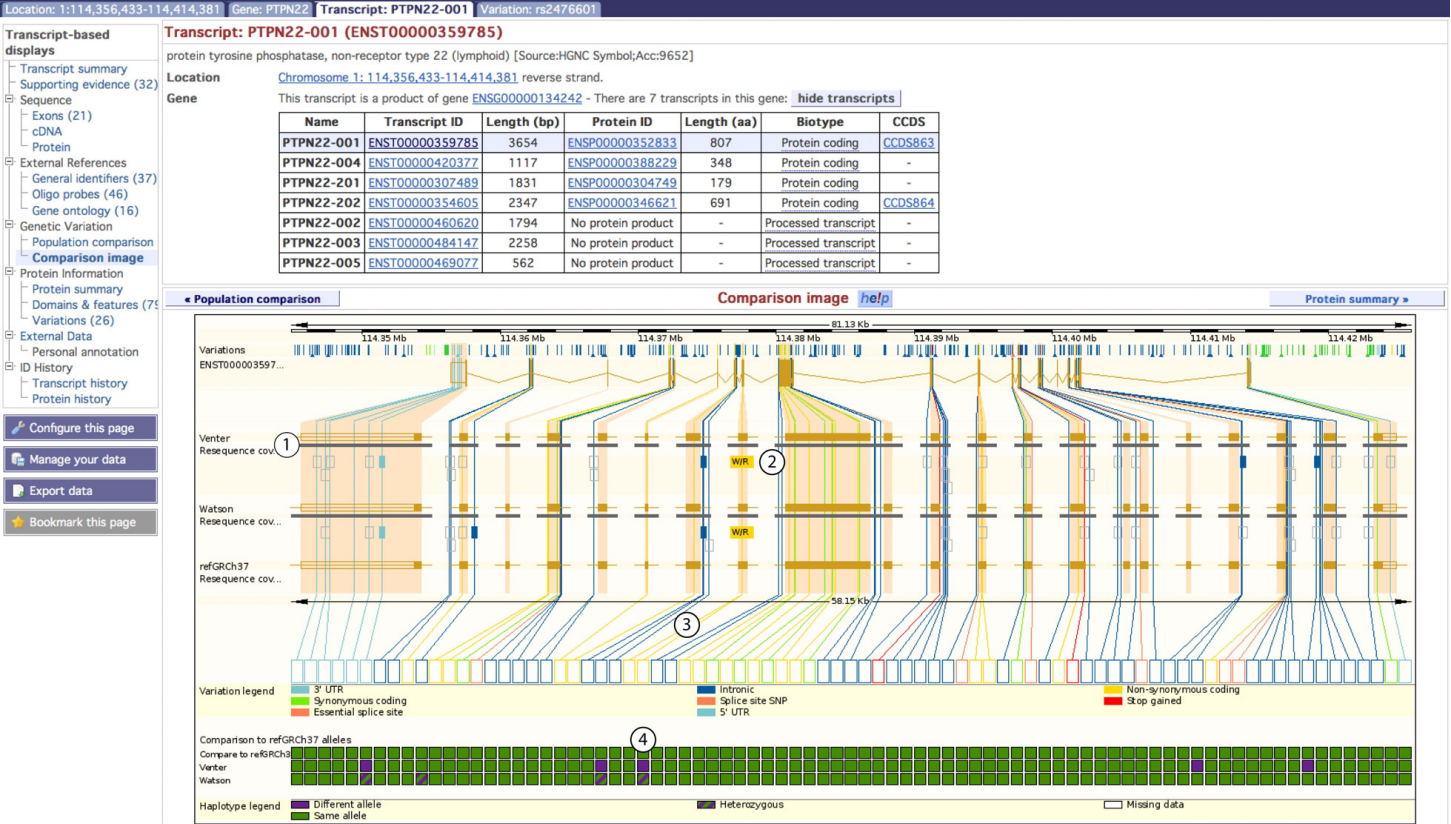
**Population comparison image**. The ENST00000359785 transcript is shown, along with a representation of the genomes of Craig Venter and James Watson and the reference sequence (GRCh37) in this region. The left hand side of the view shows links available from the transcript tab. Label 1: The genomes of Craig Venter and James Watson have high coverage in this region (i.e. at least two sequencing reads or present for both strands of the chromosome), shown by a thick grey bar. Label 2: Variants are drawn as boxes if the allele differs from the reference sequence. A non-synonymous variation is shown in yellow, with two potential amino acids indicated (tryptophan and arginine). Label 3: The nucleotides possible for this variation are displayed upon clicking the corresponding yellow box. Label 4: A table compares each allele at positions of variation. This view indicates the alleles in both Venter and Watson's genomes are different to the reference sequence allele, for this variation. Watson is heterozygous at this position, and Venter is homozygous. http://Mar2010.archive.ensembl.org/Homo_sapiens/Transcript/Population/Image?db=core;g=ENSG00000134242;r=1:114356437-114414375;t=ENST00000359785.

The gene-based variation pages described in the section above compare and collate information across alternative transcripts for the reference sequence assembly. The transcript views provide an in depth focus on one transcript, or splice isoform, across individuals, breed or strains. A list of these views and links are shown at the left of figure 5.

The transcript tab may be reached by clicking on any Ensembl transcript identifier link within the browser. All transcript identifiers (such as ENST00000359785) have the letter T immediately before the series of numbers. In the *genetic variation *section in the left hand menu of the transcript tab, the *population comparison *table and image can be found. The table lists all the variation for the transcript including details of genomic location, allele, variation source and validation status. The difference between this table and the one found in the gene tab is the ability to list variations across the genome sequences of individual humans, laboratory strains of rats and mice and breeds of dogs, chicken and other species.

The *comparison image *depicts information in the table, graphically (Figure 5). A track representing resequencing information is drawn below each transcript (Figure 5, **label 1**). A direct comparison of alleles across individuals, breeds or strains is shown (Figure 5, **label 4**).

##### cDNA sequence

**Figure 6:**
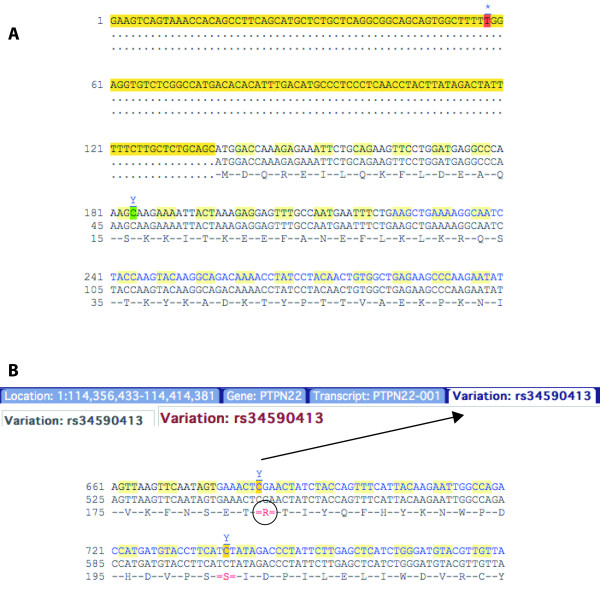
**The cDNA sequence for ENST00000359785**. **A**. The untranslated region (UTR) is highlighted in yellow. Numbering of the first line begins at 1 at the start of the UTR. Numbering of the second line starts at 1 at the beginning of the coding sequence. The third line shows line numbering and sequence of the protein. Codons are indicated by light yellow alternating with no highlight. **B**. Further down the sequence shown in A, a variation is highlighted. This variation is at position 183 in the protein sequence, 547 in the coding sequence, and 677 in the transcript sequence is shown to be rs34590413. The ID is revealed upon clicking on the IUPAC or ambiguity code above the highlighted nucleotide, which provides a link to the variation tab. The non-synonymous variations are shown by a red-coloured amino acid. Hovering over the indicated amino acid with the mouse shows the possible amino acids at that position. http://Mar2010.archive.ensembl.org/Homo_sapiens/Transcript/Sequence_cDNA?db=core;g=ENSG00000134242;r=1:114356437-114414375;t=ENST00000359785.

Variation data may be displayed on the spliced transcript sequence as in Figure 6. Select the *cDNA *link in the *sequence *section of the left hand menu in the transcript tab. Three sequence displays can be compared (cDNA, coding sequence, and protein) (Figure 6A).

In figure 6B, the red amino acid R at position 183 in the protein sequence (547 in the coding sequence, and position 677 in the transcript sequence) indicates a non-synonymous variation.

#### The location tab

The fourth and final tab described in this section contains location-based views, showing a region of the genome. View this tab by selecting it, or by clicking on any genomic coordinates-range presented in the browser. This tab was already visited in the data visualisation section of this paper as it houses the LD plots.

Variation data may be displayed in the *genomic alignments *view, highlighted where present in the sequence of the multiple species alignments. Variants can also be drawn in the *region overview *and *region in detail *views. Here, variations are displayed as vertical lines along the genome, colour-coded by the effect on the transcript. Turn variations on and off in these displays by clicking on *configure this page *and selecting the appropriate options.

##### Resequencing

**Figure 7:**
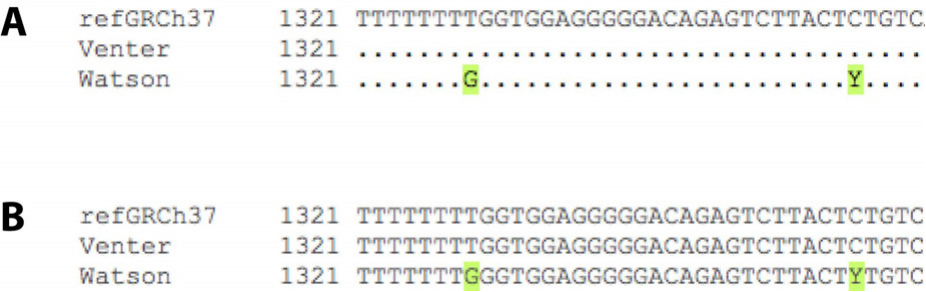
**Sequence alignments between the human reference sequence, GRCh37, and the genomes of Venter and Watson**. Variation data are highlighted in green and indicated by an IUPAC code. **A**. The default display replaces nucleotides with dots if the genome has the same allele as the reference assembly. **B**. The display can be changed by using the *matching basepairs: show all *option in the *configure this page *link. http://Mar2010.archive.ensembl.org/Homo_sapiens/Location/SequenceAlignment?db=core;g=ENSG00000134242;r=1:114382907-114387906;t=ENST00000359785.

The *genetic variation *section of the location tab shows a link to the *resequencing *alignments. These are sequence alignments across individuals, strains (for mouse, rat) or breeds (for dogs). In the example shown in figure 7, Venter and Watson's diploid genomes are compared with the reference sequence, GRCh37.

### Data export with BioMart

The BioMart tool utilises denormalised Ensembl databases and can be used to query the Ensembl core or variation databases [17]. As no programming is required, the tool may be especially useful for scientists with little computer experience. The BioMart tool is accessible from the top right of all Ensembl pages as well as from the BioMart portal at http://www.biomart.org.

Starting a BioMart query and choosing the Ensembl database (for release 56, this is listed as "Ensembl 56" from the dropdown menu, where 56 refers to the September 2009 version of Ensembl) allows gene-specific information to be exported. For example all variation for a gene can be exported in tabular format. In addition, a user may input a list of gene IDs, a region, or even an entire chromosome to mine genes and associated variations.

The Ensembl variation database may also be selected as the starting point for a BioMart query. This allows input of variation IDs, and is not contingent upon a gene annotation (i.e. intergenic regions are allowed). Furthermore, variations can be compared across populations, strains or breeds, starting with the variation database and using the "strain polymorphisms" attributes page. Export includes tabular formats such as csv, tsv, and Microsoft Excel. Alternatively, FASTA sequences can be exported.

These BioMart queries can also be run programatically using a Perl API or through Web Services. Although the programmatic scripts can be written from scratch, the simplest way to access these interfaces is to build the variation query using the BioMart website and then use the "Perl" or "XML" buttons in the toolbar to produce the appropriate API or Web Services queries.

Additional information about BioMart queries is available from the BioMart help pages at http://www.biomart.org/biomart/mview/help.html and FAQ at http://www.biomart.org/faqs.html. Video BioMart tutorials are also provided at http://www.youtube.com/watch?v=DXPaBdPM2vs&feature=channel_page.

### Data access using the API

Advanced data mining is possible through direct MySQL queries of the Ensembl public database server at ensembldb.ensembl.org or by using the Ensembl variation API [23].

### Data downloads

For convenience, Ensembl data is also available in species-specific files on the FTP site http://www.ensembl.org/info/data/ftp/index.html. This includes sequence data in FASTA format, annotation in EMBL, GENBANK and GTF format and SQL files for the databases. Resequencing data is provided for supported species in a flat file format termed EMF (Ensembl Multi Format) which is a compact, genome wide representation of the data shown in the resequencing display on the website, as described above.

## Future developments

New data is expected to greatly increase the already vast variation information in Ensembl databases. The 1000 Genomes Project is generating a large number of individual reads and variation data that will be integrated into Ensembl. There is also additional reference copy number and structural variation (CNV/SV) data planned to be incorporated from the Database of Genomic Variants [35] and other public archives that store CNV/SV data. This will also certainly result in new or enhanced web displays for these data.

The publication of many new genome wide association studies are expanding the list of alleles that can be reliably linked to disease phenotypes and both associated and causative variants will be incorporated into Ensembl as they are identified. Additionally, as part of the LRG project [36] (http://www.lrg-sequence.org/) and in collaboration with the NCBI, Ensembl is working to establish locus-specific reference sequences. These LRG (Locus Reference Genomic) sequences can be used as a framework for data exchange of annotated disease variants currently stored in diagnostic laboratories and LSDBs (Locus-Specific Databases). Both of these developments will greatly enhance the existing phenotype annotation in the Ensembl variation databases.

Other future developments include more interactive analysis of user's variation data through the Ensembl web site including an online-based upload tool for which will enable users to upload their own set of variant positions and for these to be viewable on the genome browser and for Ensembl to estimate their effects based on their relative positions to the Ensembl gene sets.

## Conclusions

Ensembl's variation resources provide a comprehensive and integrated resource for biologists and bioinformaticians to support a wide variety of research applications.

Continuously increasing biological information sources require effective and integrated bioinformatics tools and visualisation that relatively few sources are able to provide.

Ensembl's variation resources provide several advantages for users. The resources are fully integrated within to the Ensembl genome browser and with the high-quality Ensembl gene sets in order to estimate the consequences of each variant. The data is updated with each Ensembl release including automatic mapping when new genome assemblies are released, such that Ensembl's variation data may be provided in advance of a new dbSNP build. Given the breadth of species available within Ensembl, it is also easy to compare the information across many vertebrate and model organism species. Another strength of Ensembl is the availability of all its resources for free and in a variety of ways to suit a range of users as described in the availability section below. In particular, the variation API is unique among other large-scale integrative bioinformatics projects.

Finally, Ensembl is a service-focused resource. Comprehensive instructions and additional details about the project can be found at http://www.ensembl.org/info/website/help/index.html. There are video tutorials and PDFs to provide a walk-through of variation displays on the website:

http://www.ensembl.org/info/website/tutorials/index.html. Requests for assistance with any specific example or display can be addressed to helpdesk@ensembl.org.

## Availability

All Ensembl data and source code are freely available for any purpose and may be downloaded in their entirety from http://www.ensembl.org. Each Ensembl release is maintained as an archive web site for at least two years after the date of initial release (see http://www.ensembl.org/info/website/archives/index.html). Ensembl is updated approximately every two months with new data, genome assemblies, and sequenced genomes. Not every species has sufficient new data to warrant an update for each release. The current release number and month of release are shown at the bottom of every Ensembl web page.

## Authors' contributions

The database resources were created by YC, WM, DR, and FC. The variation-specific web displays were created by FC, EK, JS, BP and SB with input from all of the other authors. PMG and DS developed specific software for Ensembl variation. GS created the tutorials. FC, PF and EB defined requirements and PF provided overall project supervision. The paper was written by FC, PF, YC, GS and DS with input from all of the other authors. All authors read and approved the final manuscript.
